# The reference frame of the tilt aftereffect measured by differential Pavlovian conditioning

**DOI:** 10.1038/srep40525

**Published:** 2017-01-17

**Authors:** Yusuke Nakashima, Yoichi Sugita

**Affiliations:** 1 Department of Psychology, Waseda University, Tokyo, Japan

## Abstract

We used a differential Pavlovian conditioning paradigm to measure tilt aftereffect (TAE) strength. Gabor patches, rotated clockwise and anticlockwise, were used as conditioned stimuli (CSs), one of which (CS+) was followed by the unconditioned stimulus (UCS), whereas the other (CS−) appeared alone. The UCS was an air puff delivered to the left eye. In addition to the CS+ and CS−, the vertical test patch was also presented for the clockwise and anticlockwise adapters. The vertical patch was not followed by the UCS. After participants acquired differential conditioning, eyeblink conditioned responses (CRs) were observed for the vertical patch when it appeared to be tilted in the same direction as the CS+ owing to the TAE. The effect was observed not only when the adapter and test stimuli were presented in the same retinotopic position but also when they were presented in the same spatiotopic position, although spatiotopic TAE was weak—it occurred approximately half as often as the full effect. Furthermore, spatiotopic TAE decayed as the time after saccades increased, but did not decay as the time before saccades increased. These results suggest that the time before the performance of saccadic eye movements is needed to compute the spatiotopic representation.

Our perceptual experience of the external world is stable, despite frequent changes in the retinal images induced by eye movements. How this stability is constructed is one of the major questions in the study of the visual system. Spatiotopic representations may contribute to this stability^1^,^2^, which encodes the spatial location in external coordinates, independent of where the eyes look. Neurophysiological studies have provided evidence for spatiotopic representations. In early visual areas, such as the primary visual cortex, visual input is encoded retinotopically. Neurons in higher visual areas, including V6^3^ and the ventral intraparietal area (VIP)^4^, show activities related to spatiotopic coding. Functional magnetic resonance imaging (fMRI) studies have shown that motion signals are encoded in the spatiotopic reference frame in the human middle temporal area (hMT)^5^, and other areas including the human medial superior temporal area (hMST), the lateral occipital area (LO), and V6^6^.

One tool used to study spatiotopic coding is the aftereffect: after prolonged exposure to an adapter stimulus, such as a tilted grating, a vertical grating appears tilted in the opposite direction to the adapter. The strongest aftereffect is observed in retinotopic coordinates, i.e., when adapter and test stimuli are presented in the same retinal position. However, the tilt aftereffect (TAE) can be observed when the adapter and test stimuli are presented in a different retinal position but the same screen position, suggesting that TAE also occurs in spatiotopic coordinates^7^,^8^,^9^. Evidence for the spatiotopic aftereffect is inconsistent, with other studies reporting that TAE is exclusively retinotopic^10^,^11^. Similarly, the spatiotopic aftereffect has been reported in other features, such as motion^12^, which was subsequently disputed^13^,^14^. Spatiotopic TAE is weaker than retinotopic TAE^7^,^8^, therefore, this effect might be difficult to observe.

Here, we investigated the TAE reference frame. TAE strength was measured using a differential Pavlovian conditioning paradigm. This has several advantages since learning the relationship between the conditioned stimulus (CS) and the unconditioned stimulus (UCS) is automatic and reflexive, and does not require declarative knowledge^15^,^16^. The conditioned response (CR) may be unaffected by changes in the observer’s decisional criterion. Measurement of differential conditioning might be more objective than psychophysical measurement. Furthermore, differential eyeblink conditioning is seen in participants with no awareness of the link between the CS and the UCS^17^. Fear conditioning is observed even when the visual CS is completely suppressed from awareness^18^. Using measurement with conditioning, it might be possible to observe responses before a conscious visual experience is established.

## Results

### Experiment 1

**Figure 1:**
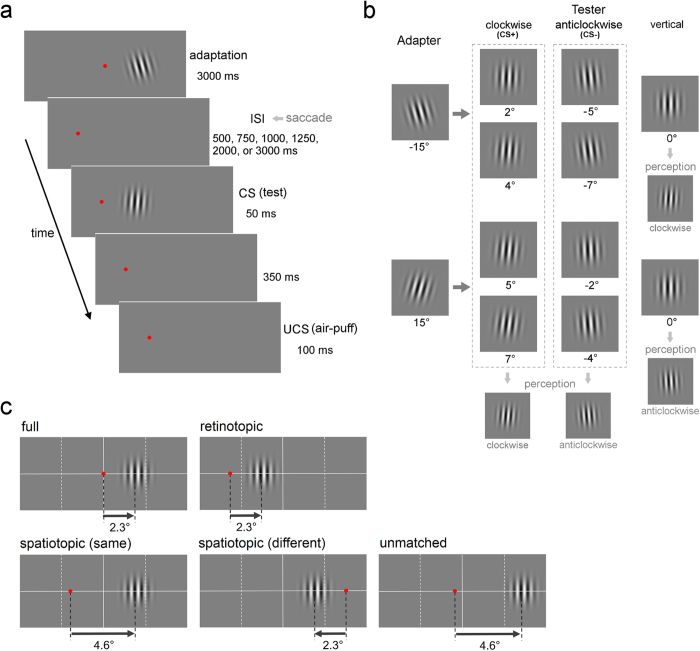
Design of Experiment 1. (**a**) Trial sequence in the retinotopic condition. The adapter stimulus was presented for 3000 ms. After the ISI, the test stimulus (CS) was presented for 50 ms. The UCS (air puff) was presented 350 ms after the CS for 100 ms. The FP was moved after the presentation of the adapter stimulus, except for the full condition. The participants were asked to perform a saccade to the new FP as soon as possible. (**b**) The four types of trials for each CS+ and CS− trial. Adapter stimuli were Gabor patches rotated clockwise and counterclockwise by 15°. Test stimuli (CS) were patches rotated clockwise and counterclockwise by 2°, 4°, 5°, and 7°. For half of the participants, the CS+ was four patches rotated clockwise and the CS− was four patches rotated counterclockwise. The test patches should appear to be tilted clockwise in all four CS+ trials and they should appear to be tilted counterclockwise in all four CS− trials. For the remaining half of the participants, the CS+ and the CS− were reversed. A vertical test patch was also presented after the clockwise and counterclockwise adapters, not followed by the UCS. After differential conditioning is acquired, eyeblink CRs are observed for the vertical patch when it appears to be tilted in the same direction as the CS+ due to TAE. (**c**) The location of the FP and the test stimulus presented after the adapter stimulus in the five reference frame conditions: the full, retinotopic, two spatiotopic, and unmatched conditions. In one spatiotopic condition, the adapter and the tester were presented in the same hemi-field. In the other spatiotopic condition, they were presented in different hemi-fields.

A Gabor patch that was rotated clockwise or anticlockwise by 15° from a vertical orientation, appeared for 3000 ms as an adapter stimulus (Fig. 1a and b). After an inter-stimulus interval (ISI), a test patch appeared for 50 ms. We altered the ISI (500, 750, 1000, 1250, 2000, and 3000 ms) to examine TAE decay time. To investigate the TAE reference frame, the experiment was conducted for five frames of reference (Fig. 1c). The fixation point (FP) was moved after presentation of the adapter stimulus, except for the full condition.

The test patches rotated clockwise and anticlockwise were used as CSs, one of which (CS+) was always followed by the UCS, whereas the other (CS−) appeared alone. The UCS was an air puff to the left eye. Each CS+ and CS− trial comprised four types (Fig. 1b). In all four types of CS+ trials, the test patches should appear to be tilted clockwise (or anticlockwise), and in all four types of CS− trials they should appear to be tilted in the opposite orientation. In addition to these CS+ and CS−, the vertical test patch also appeared for the clockwise and anticlockwise adapters. The UCS did not follow the vertical patch. After training progresses, and participants acquire differential conditioning, eyeblink CRs might be observed for the vertical patch when it appears tilted in the same direction as the CS+ due to TAE.

**Figure 2:**
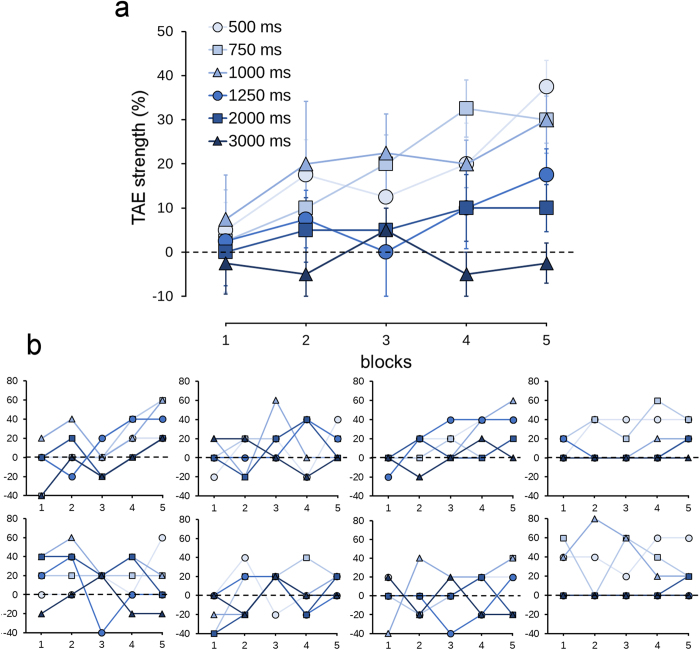
TAE strength for each block in the full condition (%). The results for the six ISI conditions are shown. (**a**) The average data across the participants. Error bars indicate the standard error. (**b**) Individual data of the eight participants.

TAE strength was measured as the percentage of CRs to the vertical test patch that appeared after the adapter patch rotated in the opposite direction to the CS+ minus the percentage of CRs to the vertical patch that appeared after the adapter patch rotated in the opposite direction to the CS−. TAE strength increased as the block progressed in the full condition (Fig. 2). As training progressed, participants exhibited eyeblink CRs for the vertical patch when the adapter patch was rotated in the opposite direction to the CS+, whereas CRs for the vertical patch were unaltered when the adapter was rotated in the opposite direction to the CS−. This indicates that CRs were elicited for the vertical patch when it appeared to be tilted in the same direction as the CS+ due to TAE.

**Figure 3:**
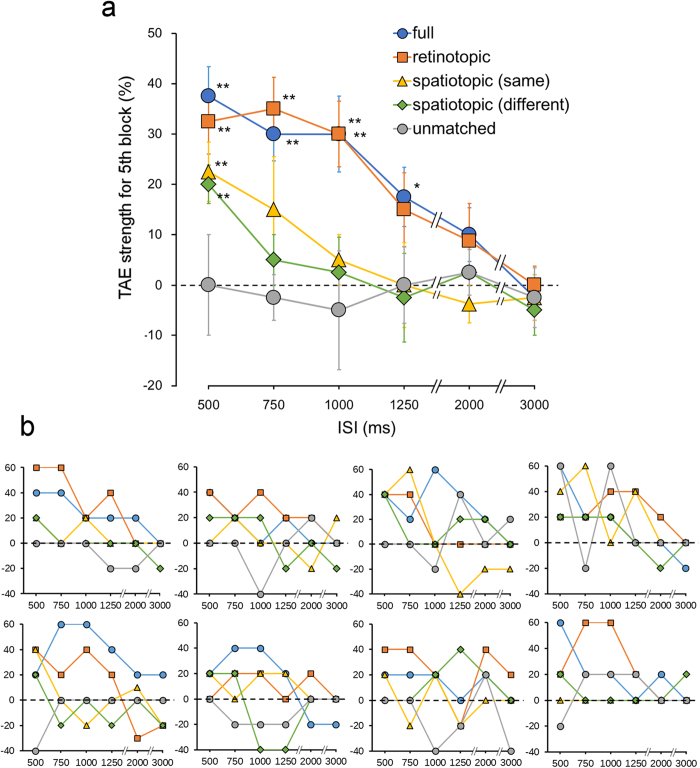
TAE strength for the fifth block (%). Data for the five reference frame conditions and for the six ISI conditions are shown. (**a**) The average data across the participants. Error bars indicate the standard error. A single asterisk indicates significance at P < 0.05 and a double asterisk indicates significance at P < 0.01 (compared with the value of 0; FDR corrected). (**b**) Individual data of the eight participants.

We compared TAE strength for the fifth block between reference frame conditions (Fig. 3). TAE was observed in the full and retinotopic conditions. TAE was also observed in the spatiotopic conditions, but not the unmatched condition. In the full condition, TAE strength was larger than zero (*t* test; The critical P value was corrected using the false discovery rate (FDR)) at 500 ms (*t*_7_ = 6.35, P = 0.0002), 750 ms (*t*_7_ = 5.61, P = 0.0004), 1000 ms (*t*_7_ = 3.97, P = 0.003), and 1250 ms ISI (*t*_7_ = 2.97, P = 0.01). In the retinotopic condition, the strength of TAE was significant at 500 ms (*t*_7_ = 5.02, P = 0.0008), 750 ms (*t*_7_ = 5.58, P = 0.0004), and 1000 ms (*t*_7_ = 4.58, P = 0.001). In the spatiotopic conditions (same and different), TAE strength was significant at 500 ms (*t*_7_ = 3.81, P = 0.003; *t*_7_ = 5.29, P < 0.0006). TAE strength at 500 ms ISI in the spatiotopic conditions were weaker than in the full (*t* test between spatiotopic (same) and full conditions: *t*_7_ = 2.51, P = 0.02; spatiotopic (different) and full conditions: *t*_7_ = 5.58, P = 0.0004), and retinotopic conditions (spatiotopic (same) and retinotopic conditions: *t*_7_ = 2.31, P = 0.027; spatiotopic (different) and retinotopic conditions: *t*_7_ = 2.82, P = 0.013). The critical P value was corrected using the FDR for comparison of these four conditions. TAE strength at 500 ms ISI was 87% in the retinotopic condition, 60% in the spatiotopic (same) condition, and 53% in the spatiotopic (different) condition, represented as a percentage of that in the full condition.

In the full, retinotopic, and spatiotopic conditions, TAE decayed with equal speed (Fig. 3). The slopes of the straight lines between 500 ms and 1000 ms ISI were not different between the conditions (*F*_3,21_ = 0.48, P = 0.70).

### Experiment 2

In Experiment 1, conditioning with the CS+ (patches rotated clockwise by 2° and 4° or rotated anticlockwise by 2° and 4°) might be generalized for the vertical test patch. It is possible that CRs to the vertical patch did not result from aftereffects but from stimulus generalization. To test this, we examined how responses to the tilted patches are generalized for patches of other orientations.

**Figure 4:**
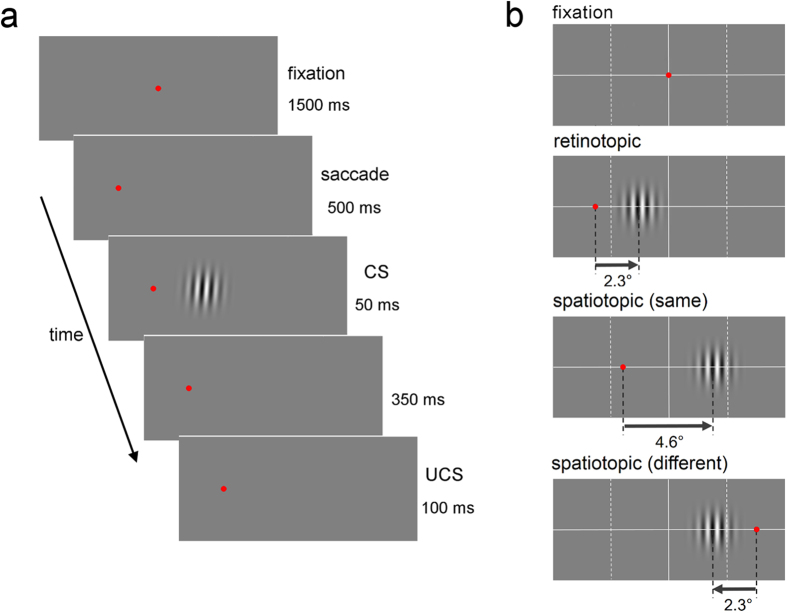
Design of Experiment 2. (**a**) Trial sequence. The FP was presented for 1500 ms. The FP moved and participants were required to perform saccades to the new FP. After 500 ms, a Gabor patch (CS) was presented for 50 ms. After 350 ms, in CS+ trials, the air puff (UCS) was presented for 100 ms. In CS− and test trials, the UCS was not presented. An adapter stimulus was not presented in Experiment 2. (**b**) The reference frame conditions: the retinotopic and two spatiotopic conditions. The top figure shows the location of the first FP. The lower three figures show the location of the second FP and the CS for each reference frame condition.

A FP appeared for 1500 ms and was then moved (Fig. 4a). After 500 ms, a Gabor patch appeared for 50 ms. An adapter stimulus was not presented. Gabor patches rotated clockwise by 2° and 4° and rotated anticlockwise by 2° and 4° were used as CSs. One orientation (clockwise or anticlockwise, CS+) was followed by the UCS, whereas the other (CS−) appeared alone. A training block consisting of 20 CS+ and 20 CS− trials was repeated three times. After training, generalization of conditioning was examined. The test block comprised patches rotated in the opposite direction to the CS+ by 1° and rotated in the same direction as the CS+ by 1°, 4°, 16°, and 64°. The UCS did not follow test patches. CS+ and CS− trials were also included in the test block to maintain conditioning.

The experiment was conducted in the retinotopic, spatiotopic (same), and spatiotopic (different) reference frame conditions to examine whether the differences in saccadic eye movement influence conditioning and its generalization (Fig. 4b).

Differential CRs for both CS+ of 2° and 4° increased as the training progressed. Differential CRs in the test block were significantly different from zero in the three conditions (retinotopic: *t*_7_ = 7.42, P = 0.0002, *t*_7_ = 8.38, P = 6e-5; spatiotopic (same): *t*_7_ = 12.83, P = 4e–6, *t*_7_ = 7.73, P = 0.0001; spatiotopic (different): *t*_7_ = 3.94, P = 0.006, *t*_7_ = 5.19, P = 0.001).

**Figure 5:**
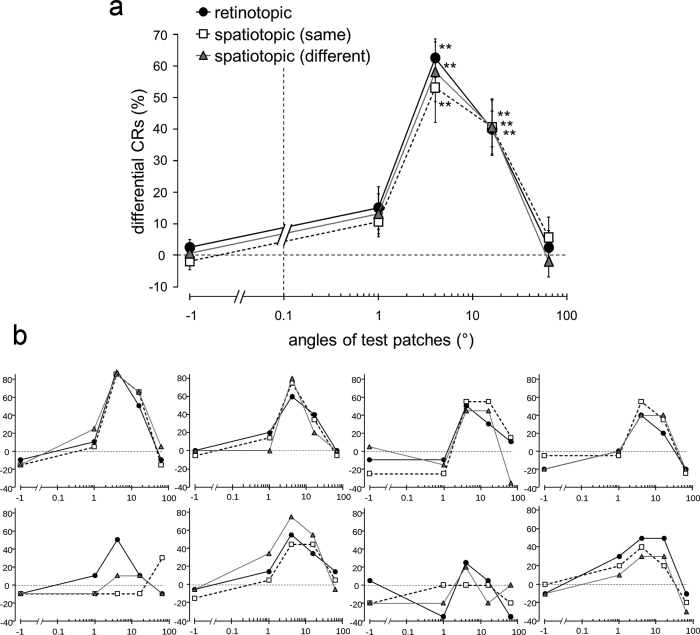
Results of Experiment 2. Differential CRs (%) for the test patches in the retinotopic, spatiotopic (same), and spatiotopic (different) reference frame conditions. Differential CRs for test patches were calculated by subtracting the percentage of CRs to the CS− from the percentage of CRs to each test patch. The x-axis is shown on a logarithmic scale. (**a**) The average data across the participants. Error bars indicate the standard error. A double asterisk indicates significance at P < 0.01 (compared with the value of 0; FDR corrected). (**b**) Individual data of the eight participants.

Differential CRs for test patches were calculated by subtracting the percentage of CRs to the CS− from the percentage of CRs to each test patch (Fig. 5). For the patch rotated in the same direction as the CS+ by 4° —that is, the same stimulus as the CS+ —large differential CRs were observed. Generalization for the patch rotated in the same direction as the CS+ by 16° was observed in the three reference frame conditions. However, generalization for the patches rotated in the same direction as the CS+ by 1° and 64°, and for the patch rotated in the opposite direction to the CS+ by 1°, was not observed in the three conditions. Differential CRs for the patches rotated in the same direction as the CS+ by 4° and 16° were significantly different from zero (retinotopic: *t*_7_ = 9.55, P = 3e-5, *t*_7_ = 6.61, P = 0.0003; spatiotopic (same): *t*_7_ = 4.52, P = 0.003, *t*_7_ = 4.42, P = 0.003; spatiotopic (different): *t*_7_ = 5.74, P = 0.0007, *t*_7_ = 4.23, P = 0.004). Differential CRs for the patches rotated in the same direction as the CS+ by 1° and 64°, and for the patch rotated in the opposite direction to the CS+ by 1°, were not significantly different from zero (retinotopic: *t*_7_ = 2.10, P = 0.07, *t*_7_ = 0.44, P = 0.67, *t*_7_ = 0.94, P = 0.38; spatiotopic (same): *t*_7_ = 2.11, P = 0.07, *t*_7_ = 0.81, P = 0.44, *t*_7_ = −0.63, P = 0.55; spatiotopic (different): *t*_7_ = 1.93, P = 0.10, *t*_7_ = −0.36, P = 0.73, *t*_7_ = 0.20, P = 0.85). The critical P value was corrected using the FDR in each condition. We also compared differential CRs between the three reference frame conditions. However, there were no differences (main effect of the reference frame conditions: *F*_2, 14_ = 0.69, P = 0.52; main effect of the test patches: *F*_4, 28_ = 28.33, P < 0.001; interaction effect: *F*_8, 56_ = 0.38, P = 0.93).

### Experiment 3

TAE in the retinotopic and spatiotopic reference frames decayed with equal speed in Experiment 1. It has been reported that TAE in the spatiotopic reference frame takes time to build up^9^. Spatiotopic TAE was observed only when the saccade target appeared for at least 500 ms before saccades, and the magnitude of spatiotopic TAE increased as the display time of the saccade target increased. The present results were apparently inconsistent with these findings. In the present study, the target display time was not manipulated before saccades but the ISI varied between the adapter offset and the tester onset. The FP was moved to a new location at the same time as the adapter offset and participants were required to quickly perform saccades. However, the participants in Zimmermann *et al*.^9^ kept looking at the same FP for a certain amount of time after the adapter offset. This might account for the discrepancy in results. In Experiment 3, we conducted a similar experiment in Experiment 1, manipulating the display time of the saccade target before initiating saccades.

**Figure 6:**
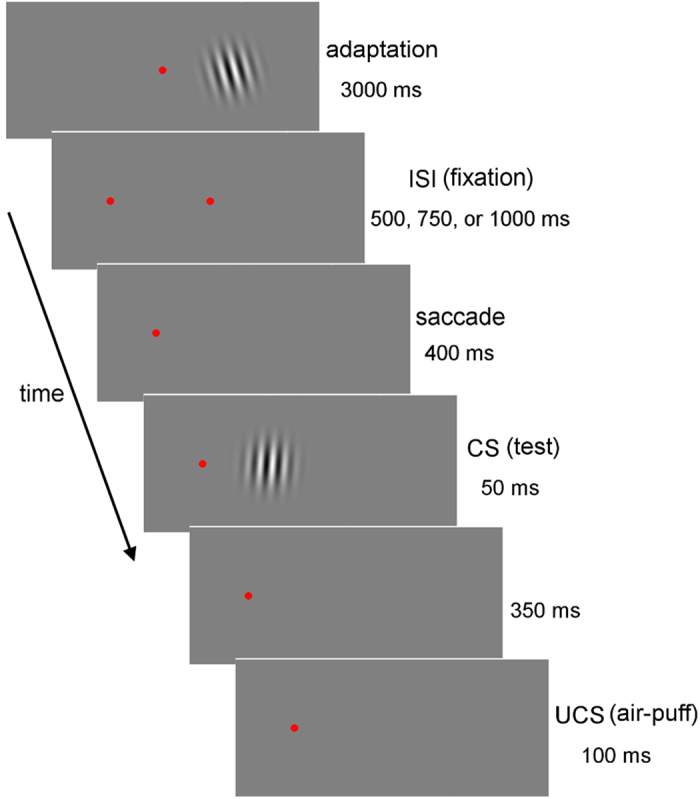
Trial sequence for the retinotopic condition in Experiment 3. After an adaptation period of 3000 ms, the target was presented while the FP still appeared. After a duration of ISI, the FP disappeared and the participants were required to perform a saccade to the target. The test stimulus (CS) was presented 400 ms after extinction of the FP. The UCS was presented 350 ms later. The duration of ISI was 500, 750, or 1000 ms.

The methods in Experiment 3 was the same as Experiment 1 except for the timing of saccades. After the adapter stimulus was presented, the saccade target appeared while the FP remained (Fig. 6). After ISI, the FP disappeared and participants were required to perform saccades. A test stimulus appeared 400 ms after extinction of the FP. The ISI was 500, 750, or 1000 ms. Retinotopic and spatiotopic (different) conditions were conducted.

**Figure 7:**
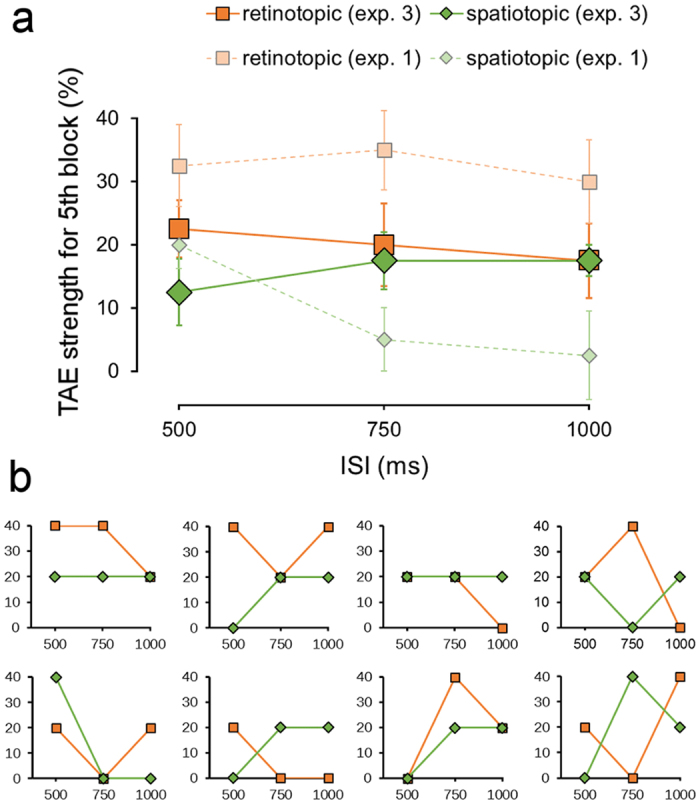
TAE strength (%) for the fifth block for the retinotopic and the spatiotopic (different) conditions in Experiment 3. (**a**) The average data across the participants. The results in Experiment 1 were replicated. Error bars indicate the standard error. (**b**) Individual data of the eight participants.

We compared TAE strength in the fifth block between the retinotopic and spatiotopic conditions (Fig. 7). Consistent with Experiment 1, TAE decayed gradually in the retinotopic condition. However, there was no decay in the spatiotopic condition. The slope of the straight line between 500 ms and 1000 ms ISI in the retinotopic condition was not different between Experiment 1 and Experiment 3 (*t*_14_ = 0.20, P = 0.42). However, the slope for the spatiotopic (different) condition in Experiment 3 was larger than that in Experiment 1 (*t*_14_ = 2.29, P = 0.02). There was no difference between the retinotopic and spatiotopic conditions in Experiment 3 (*t*_7_ = 1.18, P = 0.14).

## Discussion

The present study investigated the TAE reference frame, using a differential Pavlovian conditioning paradigm. Eyeblink CRs were elicited for the vertical test patch when it appeared tilted in the same direction as the CS+ because of TAE. TAE was observed both when the adapter and the test stimuli were presented, not only in the same retinotopic position, but also in the same spatiotopic position, although the spatiotopic TAE was weak—it occurred approximately half as often as the full effect. TAE in the full, retinotopic, and spatiotopic conditions decayed with equal speed.

TAE occurs in retinotopic and spatiotopic reference frames^7^,^8^,^9^. However, TAE may also be exclusively retinotopic^10^,^11^. The strength of spatiotopic TAE is very weak, approximately half the strength of full TAE^7^,^8^, thus spatiotopic TAE might be difficult to observe and is presumably susceptible to response and decisional biases. Observers can easily change their psychometric functions by introducing a response bias or change in criterion^19^. It has been argued that high level aftereffects, such as face aftereffects^20^ and crossmodal aftereffects^21^,^22^, might not be caused by perceptual encoding changes but by changes in the criteria used for decision-making^23^. It was also reported that, when using a relatively bias-free two alternative forced choice procedure, no spatiotopic TAE occur. This suggests that spatiotopic TAE is not a perceptual effect, but instead occurs owing to changes in decisional criteria^24^.

Pavlovian conditioning might occur independent from consciousness^17^,^18^,^25^,^26^. Differential conditioning was observed in participants who showed no conscious awareness of the link between the CS and the UCS^17^. Fear conditioning can be acquired even when the visual CS is completely suppressed from awareness by continuous flash suppression^18^. Moreover, conditioning is observed even in patients in a vegetative or minimally conscious state^25^, and in subjects during sleep^26^. Measurement of Pavlovian conditioning might reveal the sensory processing that occurs prior to decision-making. Conditioned eyeblink responses were initiated without intent and mediated by a low level learning mechanism, for which the loci responsible include the cerebellum and the brainstem^27^. In contrast, subjective judgment, such as button press, is processed through the frontal cortex. Compared with subjective judgments, the measurement of conditioning may be more objective and less influenced by decisional biases.

It is possible that CRs to the vertical patch did not result from aftereffects but from stimulus generalization. Thus, we examined whether conditioning, with patches rotated clockwise or anticlockwise, is generalized for patches of other orientations in Experiment 2. After conditioning was acquired with the patches rotated by 2° and 4°, conditioning was not generalized for the patches rotated in the same direction as the CS+ by 1° and rotated in the opposite direction to the CS+ by 1°. This suggests that CRs to the vertical patch presented after the adapter stimulus did not occur because of stimulus generalization but instead because of the aftereffect. Generalization occurred when the patch was rotated in the same direction as the CS+ by 16°, but not when patches were rotated in the same direction as the CS+ by 1° and rotated in the opposite direction to the CS+ by 1°. This indicates that generalization occurs for stimuli that appear to be tilted in the same direction as a CS+, but not for stimuli that appear to be another orientation, despite the angle difference between a CS+ and a test stimulus being slight. Similar results were obtained in the retinotopic, spatiotopic (same), and spatiotopic (different) reference frame conditions, indicating that the differences in saccades do not influence conditioning and its generalization.

TAE in the retinotopic and spatiotopic reference frames decayed with equal speed in Experiment 1. It has been reported that TAE in the spatiotopic reference frame takes time to build up^9^. Spatiotopic TAE was observed only when the saccade target was displayed for at least 500 ms before initiating saccades. Moreover, the magnitude of spatiotopic TAE increased as the display time of the target increased from 0 to 1000 ms, although retinotopic TAE decayed slightly. The present results contradict this finding. The difference in the methods between Zimmermann *et al*.^9^ and the present study is the timing of saccades. Zimmermann *et al*. manipulated the display time of the target before initiating saccades while we varied the ISI after the gaze changed. This difference might be due to the discrepancy in the time course of spatiotopic TAE. In Experiment 3, in line with Zimmermann *et al*., spatiotopic TAE did not decay, unlike the results in Experiment 1. This indicates that spatiotopic TAE decays as the time after initiating saccades increases, but does not decay or develop as the display time of the target before the initiation of saccades increases up to 1000 ms. This suggests that a sufficient display time of the saccade target position before initiating saccades, not the time after initiating saccades, is needed to compute the spatiotopic representation.

The spatiotopic effects were observed in two different conditions. In one condition, the adapter and test stimuli were presented in the same hemi-field, whereas in the other condition they were presented in different hemi-fields, suggesting that spatiotopic TAE transfers strongly between the two hemispheres. The strength of the effect was similar between the two spatiotopic conditions, which were different in the hemi-field where the test stimulus appeared in the contingent motion aftereffect^28^. Strong inter-hemispheric transfer might occur in spatiotopic aftereffects.

## Methods

All participants had normal or corrected-to-normal vision, and were naïve to the purpose of the experiment. Informed consent was obtained for all participants. Experiments were approved by the local ethics committee of Waseda University, and performed in accordance with the Declaration of Helsinki.

### Experiment 1

#### Participants

Eight participants (21–25 years old) took part in this experiment.

#### Stimuli

Two-dimensional visual stimuli were presented on a 24-inch CRT display (800 × 600 pixel resolution, refresh rate of 60 Hz) with a viewing distance of 1 m. Both adaptation and test stimuli were sinusoidal luminance modulations with a spatial frequency of 4.0 cycles per degree, maximum contrast, linear envelop, phase of 0°, and radius of 2.0° presented on a uniform grey background (24.74 cd/m^2^).

#### Procedure

Participants were asked to keep looking at a red FP (27.95 cd/m^2^ and 0.3° in diameter) in a dark room. The FP was presented in the screen centre. A Gabor patch that was rotated clockwise or anticlockwise by 15° from a vertical orientation appeared for 3000 ms as an adapter stimulus. The centre of the adapter stimulus was 2.3° to the right of the FP. After an ISI, a test patch was presented 2.3° to the right of the FP. We altered the ISI (500, 750, 1000, 1250, 2000, and 3000 ms) to examine the TAE decay time. The duration of the test patch was 50 ms.

To measure TAE strength we used differential conditioning. The test patches that were rotated clockwise and anticlockwise were used as CSs, one of which (CS+) was always followed by the UCS, whereas the other (CS−) was presented alone. For half of the participants, the CS+ involved patches rotated clockwise and the CS− involved patches rotated anticlockwise. For the remaining half, the CS+ and the CS− were reversed. The UCS was a 100 ms, 5 psi air puff. It was delivered to the left eye via flexible plastic tubing terminating in a 1 mm nozzle placed 45° left of the left eye. The distance between the nozzle tip and the cornea was approximately 3 cm. In the CS+ trials, the UCS was presented 400 ms after the CS onset.

Eye blinks were recorded using pairs of electromyographic (EMG) electrodes (4 mm Ag-AgCl). The electrodes were placed on the orbicularis palpebrarum muscle below each eye with a ground electrode on the forehead. EMG data were continuously recorded at 1 kHz. An eyeblink occurring from 100 ms after CS onset to UCS onset, the amplitude of which was equal to or greater than 10% of the average amplitude in response to the UCS presentation in the same session, was counted as an eyeblink CR.

To monitor eye movements, electrooculography (EOG) was measured with two electrodes placed near the outer canthi of both eyes. We evaluated whether the participants performed the correct saccades response from the first FP to the second FP. Trials in which saccades were performed at the wrong time were rejected, i.e., saccades initiated before the offset of the adapter stimulus or occurring after the onset of the test stimulus. Trials in which EOG amplitude was smaller or larger than that for a correct saccade response were also rejected. To confirm that saccades responses of an incorrect size could be detected by EOG amplitude, we compared EOG and gaze position data measured by an eye tracker (Supplementary Fig. S1). Participants were trained for the saccade response in each reference frame condition before the experiment.

Each CS+ and CS− trial comprised four different types of trials. For half of the participants, in the CS+ trials, test patches that were rotated clockwise by 2° and 4° were presented after the presentation of the adapter patch rotated anticlockwise, and test patches that were rotated clockwise by 5° and 7° were presented after the presentation of the adapter patch rotated clockwise. In all four types of CS+ trials, the test patches should appear to be tilted clockwise. In the CS− trials, the same four types of trials were used as the CS+ trials except that the clockwise and anticlockwise rotations were reversed. In all four types of CS− trials, the test patches should appear to be tilted anticlockwise. For the remaining half of the participants, the CS+ and the CS− were reversed. In addition to these four CS+ and four CS−, the vertical test patch was also presented for the clockwise and anticlockwise adapters. The UCS did not follow the vertical patch. After training progresses and participants acquire differential conditioning, eyeblink CRs might be observed for the vertical patch when it appears to be tilted in the same direction as the CS+ due to TAE. TAE strength was measured as the percentage of CRs to the vertical test patch that were presented after the presentation of the adapter patch rotated in the opposite direction to the CS+ minus the percentage of CRs to the vertical patch that were presented after the presentation of the adapter patch rotated in the opposite direction to the CS−.

The experiment comprised 250 trials. Each block of 50 trials included 20 CS+, 20 CS−, and 10 vertical patch trials. Trials were presented randomly, with no more than two trials of the same type in a row.

In the experimental condition mentioned above, the test patch was presented not only in the same retinal location, but also in the same screen location as the adapter patch (full condition). To examine the TAE reference frame, we tested the other four reference frame conditions. In all conditions, the adapter stimulus was presented 2.3° to the right of the FP, which was presented in the screen centre, and then the adapter disappeared and the FP was moved, except in the full condition. The participants were asked to quickly perform a saccade to the new FP. After the ISI, the test stimulus was presented. In the retinotopic condition, the FP was presented 5.2° to the left of the screen centre and the test stimulus was presented 2.3° to the right of the FP so that the adapter and test stimuli were presented in the same retinal location but at a different screen location. The spatiotopic condition was tested for two different FPs, where the adapter and test stimuli were presented at the same screen location but in different retinal locations. In one spatiotopic condition, the FP was presented 2.3° to the left of the screen centre and the test stimulus was presented 4.6° to the right of the FP. In the other spatiotopic condition, the FP was presented 4.6° to the right of the screen centre and the test stimulus was presented 2.3° to the left of the FP. In the first spatiotopic condition, the adapter and test stimuli were presented in the same hemi-field, whereas in the second spatiotopic condition they were presented in different hemi-fields. In the unmatched condition, the FP was presented 0.6**°** to the left of the screen centre and the test stimulus was presented 4.6° to the right of the FP so that the adapter and test stimuli were presented in different retinal and screen locations. Each participant underwent another 30 sessions (the five reference frame conditions × the six ISI conditions). The reference frame conditions were tested across different days and in a random order between participants. The ISI conditions in the reference frame condition were tested on a day with a break (at least 5 minutes).

### Experiment 2

#### Participants

Eight participants (21–26 years old) took part in this experiment.

#### Stimuli

The same stimuli as Experiment 1 were presented on a uniform grey background (26.12 cd/m^2^).

#### Procedure

A red FP (31.04 cd/m^2^) was presented in the screen centre for 1500 ms and then the FP was moved. After 500 ms, a Gabor patch was presented for 50 ms. An adapter stimulus was not presented.

Gabor patches rotated clockwise by 2° and 4° and anticlockwise by 2° and 4° were used as CSs. One orientation (clockwise or anticlockwise, CS+) was followed by an air puff (UCS), whereas the other (CS−) was presented alone. A training block consisting of 20 CS+ and 20 CS− trials was repeated three times. After training, a test was conducted to examine the generalization of conditioning. The test block was comprised of patches rotated in the opposite direction to the CS+ by 1° and rotated in the same direction as the CS+ by 1°, 4°, 16°, and 64°. The UCS did not follow test patches. Each test patch was presented five times. Twenty CS+ and twenty CS− trials were also included in the test block to maintain conditioning.

The experiment was conducted in the retinotopic, spatiotopic (same), and spatiotopic (different) reference frame conditions to examine whether differences in saccadic eye movements between the conditions influence conditioning and its generalization. The locations of the FP and stimulus in each condition were the same as Experiment 1, although the adapter stimulus was not presented.

### Experiment 3

#### Participants

Eight participants (21–27 years old) took part in this experiment.

#### Stimuli

The same stimuli as in Experiment 1 were presented on a uniform grey background (25.87 cd/m^2^).

#### Procedure

Experiment 3 was the same as Experiment 1, except for the timing of saccades. An FP (29.54 cd/m^2^) was presented in the screen centre and an adapter stimulus appeared to the right of the FP. After an adaptation period of 3000 ms, the target was presented while the FP appeared. After an ISI, the FP disappeared and participants were required to quickly perform a saccade to the target. The test stimulus (CS) was presented 400 ms after extinction of the FP. The UCS was presented 350 ms after CS offset. The duration of ISI was 500, 750, or 1000 ms. Retinotopic and spatiotopic (different) conditions were conducted.

## Additional Information

**How to cite this article:** Nakashima, Y. and Sugita, Y. The reference frame of the tilt aftereffect measured by differential Pavlovian conditioning. *Sci. Rep.* **7**, 40525; doi: 10.1038/srep40525 (2017).

**Publisher's note:** Springer Nature remains neutral with regard to jurisdictional claims in published maps and institutional affiliations.

## Supplementary Material

Supplementary Information
