# Supplementary material for: The reference frame of the tilt aftereffect measured by differential Pavlovian conditioning
Source: Sci Rep. 2017 Jan 17;7:40525. doi: 10.1038/srep40525 (PMC5240094; doi:10.1038/srep40525)
Supplement: Supplementary Information [file srep40525-s1.pdf]

Supplementary Information

The reference frame of the tilt aftereffect measured by differential Pavlovian conditioning.

Yusuke Nakashima* and Yoichi Sugita

Department of Psychology, Waseda University, Tokyo, Japan

Supplementary Figure:

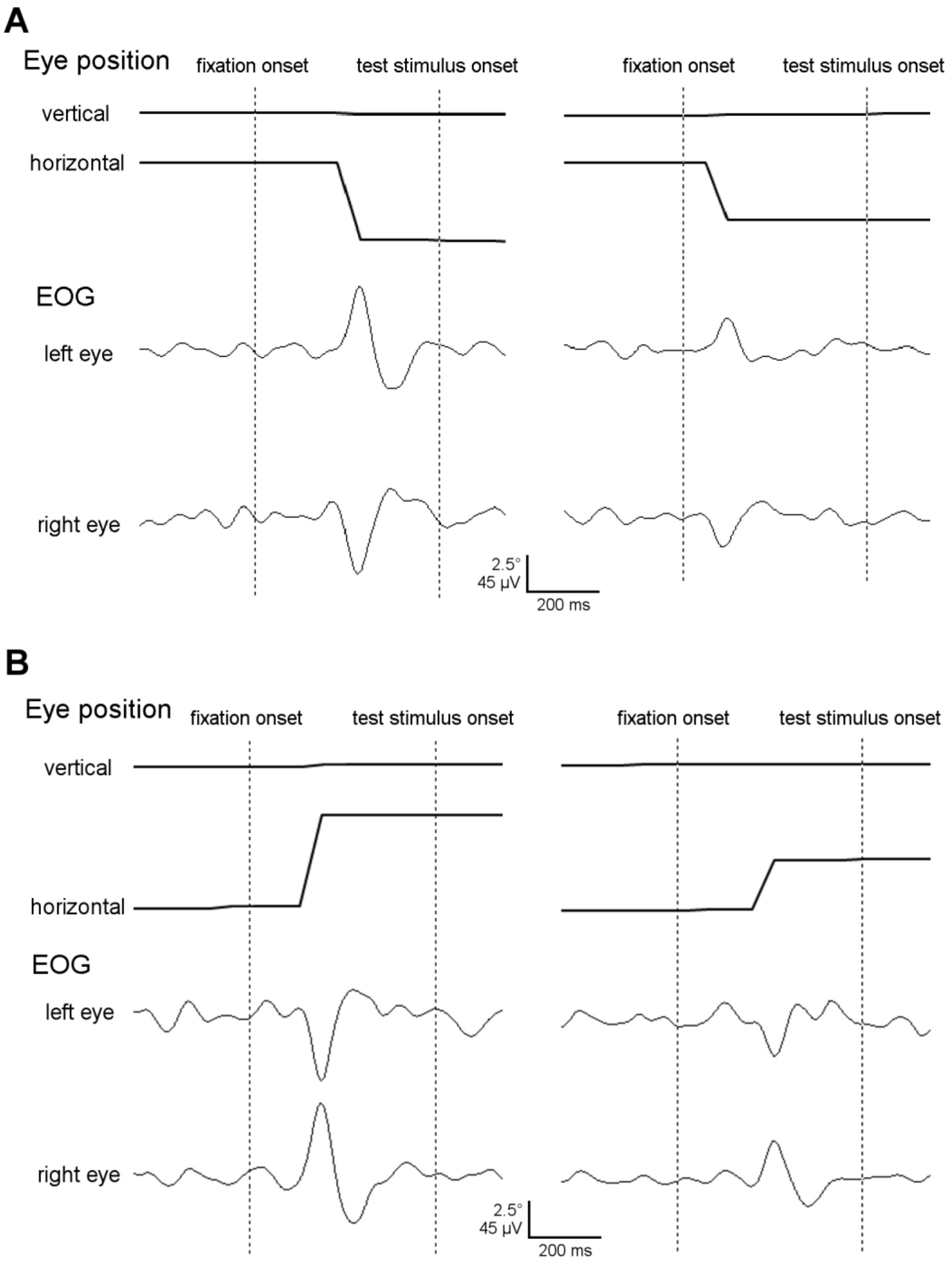

Figure S1. Gaze position and electrooculography (EOG) data in trials of Experiment 1. To confirm that incorrect saccades can be detected by EOG, the two data were measured together. The gaze position was measured by recording the movements of the left eye with using an infrared-video-based eye tracker (Quick Glance 3, EyeTech Digital Systems, sampling at 30 Hz). The two vertical dotted lines indicate the onset of the second fixation

(presented after the adapter stimulus) and the onset of the test stimulus (CS). (A) The data for trials in the retinotopic condition. The left figure shows the data for a correct saccade which landed at the second fixation. The right figure shows the data for an incorrect saccade which landed at the center of the test stimulus. The incorrect saccade can be detected by the EOG amplitude. (B) The data for trials in the spatiotopic (different) condition. The left figure shows the data for a correct saccade and the right figure shows the data for an incorrect saccade which landed at the center of the test stimulus. The incorrect saccade can also be detected by the EOG amplitude in this condition.
